# Editorial: Novel mechanisms involved in aging and neurodegeneration: Seeking potential therapeutic targets for neurodegenerative diseases

**DOI:** 10.3389/fnagi.2022.1104647

**Published:** 2022-12-09

**Authors:** Mario Sanhueza, Yasmina Manso, Claudio Soto, Natalia Salvadores

**Affiliations:** ^1^Center for Resilience, Adaptation and Mitigation, Universidad Mayor, Temuco, Chile; ^2^Department of Cell Biology, Physiology, and Immunology, Faculty of Biology, Institute of Neurosciences, Universitat de Barcelona, Barcelona, Spain; ^3^Centro de Investigación Biomédica en Red Enfermedades Neurodegenerativas, Instituto de Salud Carlos III, Madrid, Spain; ^4^Mitchell Center for Alzheimer's Disease, Department of Neurology, McGovern Medical School, The University of Texas Health Science Center at Houston, Houston, TX, United States

**Keywords:** aging, neurodegeneration, therapeutic targets, protein aggregation, Alzheimer's disease, Parkinson's disease

During the past decades, the world's demographics have changed considerably, with an aging global population that is expected to keep rising due to constant progress in biomedical research. Aging constitutes the main risk factor for chronic diseases including neurodegenerative conditions and therefore, the incidence of brain diseases associated with aging is growing, constituting a major health issue worldwide (GBD 2017 US Neurological Disorders Collaborators et al., 2021). Although neurodegenerative disorders have different clinical manifestations, several pathogenic processes are common to these diseases. These include cerebral accumulation of misfolded protein aggregates, inflammation, altered proteostasis, changes in response to cellular stress, altered intercellular communication, damage to the synaptic network, mitochondrial dyshomeostasis, and cellular senescence (Habib et al., 2018). This Research Topic comprises 12 articles, involving 98 authors across the globe, which describe new evidence connecting molecular, cellular, and physiological mechanisms with the development of neurodegenerative disorders.

Sleep disruption is a common feature in several neurodegenerative disorders (Mander et al., 2016). The orexinergic system is essential for sleep-wake state regulation, and dysregulation of this system is thought to contribute to neurodegenerative disease pathogenesis (Asai et al., 2009; Fatemi et al., 2016; Liguori, 2017). A comprehensive review by Wang et al. describes evidence for the role of the orexinergic system in Alzheimer's disease (AD), Parkinson's disease (PD), Huntington's disease (HD), and multiple sclerosis (MS). The authors of this manuscript highlight the orexinergic system as a potential target for disease treatment.

AD represents the most common neurodegenerative disease (Alzheimer's Association, 2022). This devastating neurological condition has no cure yet, and many efforts are currently underway to identify novel targets for AD treatment. In this context, the N-methyl-D-aspartate (NMDA) receptor is a critical molecule for synaptic plasticity and cognitive function, both altered in AD. Using the APP/PS1 model of AD, Xu et al. found alterations in NMDA receptor function, paralleled by reduced expression of synaptic proteins, decreased dendritic complexity, and dendritic spine density. The authors conclude that these changes might underlie the electrophysiological and synaptic plasticity impairments observed in the hippocampus of this model, which may in turn lead to the age-related cognitive deficits observed. The presence of the APOEε4 and Trem2^*^R47H alleles, constitute two of the most powerful risk factors for late-onset AD (Cacace et al., 2016). Using a novel mouse model of AD expressing these genes, Kotredes et al. carried out a compelling characterization, identifying behavioral, transcriptomic, metabolic, and neuropathological alterations, including disruption of glycolytic metabolism and vascular perfusion in this new model.

Cerebral vascular disease is a known contributor to vascular cognitive impairment (VCI) and AD (Gorelick et al., 2011). Investigating the possible mechanisms involved, Li et al. used a model in which Tg-SwDI mice were subjected to bilateral common carotid stenosis (BCAS), a well-characterized murine model of VCI. The researchers observed that mice that underwent BCAS exhibited impaired glymphatic drainage as well as reduced vascular pulsation unveiling potential targets for VCI treatment. Another feature shared by vascular dementia (VaD) and AD is neuronal cytoskeleton disruption characterized by microtubule destabilization (Mukaetova-Ladinska et al., 2015; Zhang et al., 2015). In this context, Santiago-Mujika et al. summarized current knowledge on posttranslational modifications of tubulin that affect microtubule dynamics and discuss why restoring the microtubule network might be critical to stopping the progression of neurodegeneration in the context of AD and VaD.

Diagnosis of mild cognitive impairment (MCI) is crucial for identifying subjects at high risk of AD. In this line, the development of biomarkers for MCI has been the focus of intense research, which is the issue reviewed by Ogonowski et al.. In this manuscript, the authors cover literature regarding diagnostic criteria in MCI, traditional and new biomarkers in the diagnosis of MCI and then focus on microRNAs as potential biomarkers. This systematic review identified five miRNA expression profiles, which may be exploited as fluid biomarkers of early-stage AD.

PD is the second most prevalent neurodegenerative disease, characterized by progressive loss of motor function. Current treatments can only slightly decrease the symptoms but do not stop the process of neurodegeneration (Kalia and Lang, 2015). Using a novel *C. elegans* PD model expressing α-Synuclein in DA neurons, Vozdek et al. performed a GWAS for a functional search of PD risk genes. The researchers identified several modulators of α-Synuclein toxicity and provided the basis for follow-up studies aimed at further characterizing the role of these genes in PD, which may represent new potential therapeutic targets for synucleinopathies. Growing evidence has shown the benefits of exercise in PD patients (Fox et al., 2018). Yet, the potential mechanisms underlying exercise-associated improvements in PD remain unknown, which is the focus of the study by Shi et al.. In this article, the authors show that exercise intervention alleviates motor deficits in a rat PD model based on 6-OHDA administration. Researchers provide evidence that exercise treatment regulates brain plasticity in this PD model by suppressing the overexcitability of local field potentials (LFP) and minimizing spike-LFP synchronization in the motor cortex. The loss of parvalbumin-positive (PV+) neurons in the substantia nigra pars reticulata (SNR) has been demonstrated in animal models of PD and postmortem PD brains (Hardman et al., 1996; Pamukcu et al., 2020). To investigate the progression of PV+ neuron loss, Zheng et al. used a transgenic PD mouse model previously developed by the research group (Jiao et al., 2020). Histopathological analyses of these aged mice revealed increased accumulation of α-synuclein in the SNR which was concomitant with neurodegeneration and progressive apoptosis activation, providing evidence of the interplay between Tau and α-synuclein in the mechanism of neuronal loss.

Given the fact that amyloid aggregates constitute a histopathological hallmark of most neurodegenerative diseases, the role of amyloids in the development of these disorders has been widely studied (Soto and Pritzkow, 2018). In this line, many studies have centered on the molecular properties of these proteins. In his review, Diaz-Espinoza presents state-of-the-art progress on the amyloid structures reported for amyloid-β, tau, and α-synuclein, which has been facilitated by the use of solid-state nuclear magnetic resonance and cryogenic-electron microscopy. The author further discusses the potential of these models for the design of novel therapies against neurodegenerative diseases.

The study by Zhao et al. uncovers evidence of NLRP3 as a modulator of hyperphosphorylated Tau toxicity. Employing a traumatic brain injury and a tau hyperphosphorylation mouse model, the researchers were able to demonstrate an increase in NLRP3 in response to tau overexpression, which was associated with neurodegeneration. Moreover, NLRP3 knockout prevented the upregulation of HMGB1 and pTau levels. Interestingly, treatment with an HMGB1 inhibitor resulted in improvements in the cognitive performance of tau-overexpressing mice, unveiling HMGB1 as a potential target for tauopathies.

Myotonic dystrophy type 1 (DM1) is a multisystem disorder that affects muscles, as well as the eye, heart, endocrine system, and central nervous system (Nguyen and Campbell, 2016). The manuscript by Liu et al. summarizes the evidence around the central nervous system deficits in DM1, highlighting potential therapeutic strategies for the disease.

The studies presented in this Research Topic expand the current understanding of the mechanisms contributing to neurodegeneration. Importantly, the increased knowledge regarding the pathways involved in the processes underlying neurodegenerative diseases will be key to uncovering new therapies for brain disorders.

## Author contributions

MS, YM, CS, and NS have contributed to manuscript writing and editing. MS coordinated, reviewed, and checked the final version. All authors have made a substantial intellectual contribution to this manuscript and approved it for publication.
